# Locus Coeruleus Integrity from 7 T MRI Relates to Apathy and Cognition in Parkinsonian Disorders

**DOI:** 10.1002/mds.29072

**Published:** 2022-05-16

**Authors:** Rong Ye, Claire O'Callaghan, Catarina Rua, Frank H. Hezemans, Negin Holland, Maura Malpetti, P. Simon Jones, Roger A. Barker, Caroline H. Williams‐Gray, Trevor W. Robbins, Luca Passamonti, James Rowe

**Affiliations:** ^1^ Department of Clinical Neurosciences and Cambridge University Hospitals NHS Trust University of Cambridge Cambridge United Kingdom; ^2^ Brain and Mind Centre and School of Medical Sciences Faculty of Medicine and Health, University of Sydney Sydney Australia; ^3^ Department of Psychiatry University of Cambridge Cambridge United Kingdom; ^4^ MRC Cognition and Brain Sciences Unit University of Cambridge Cambridge United Kingdom; ^5^ Department of Clinical Neurosciences, John van Geest Centre for Brain Repair University of Cambridge Cambridge United Kingdom; ^6^ Wellcome Trust—Medical Research Council Stem Cell Institute University of Cambridge Cambridge United Kingdom; ^7^ Department of Psychology University of Cambridge Cambridge United Kingdom; ^8^ Behavioural and Clinical Neuroscience Institute University of Cambridge Cambridge United Kingdom; ^9^ Istituto di Bioimmagini e Fisiologia Molecolare Consiglio Nazionale delle Ricerche Cefalù Italy

**Keywords:** locus coeruleus, Parkinson's disease, progressive supranuclear palsy, cognition, apathy, magnetization‐transfer imaging, 7 T magnetic resonance imaging, noradrenaline

## Abstract

**Background:**

Neurodegeneration in the locus coeruleus (LC) contributes to neuropsychiatric symptoms in both Parkinson's disease (PD) and progressive supranuclear palsy (PSP). Spatial precision of LC imaging is improved with ultrahigh field 7 T magnetic resonance imaging.

**Objectives:**

This study aimed to characterize the spatial patterns of LC pathological change in PD and PSP and the transdiagnostic relationship between LC signals and neuropsychiatric symptoms.

**Methods:**

Twenty‐five people with idiopathic PD, 14 people with probable PSP‐Richardson's syndrome, and 24 age‐matched healthy controls were recruited. Participants underwent clinical assessments and high‐resolution (0.08 mm^3^) 7 T‐magnetization‐transfer imaging to measure LC integrity in vivo. Spatial patterns of LC change were obtained using subregional mean contrast ratios and significant LC clusters; we further correlated the LC contrast with measures of apathy and cognition, using both mixed‐effect models and voxelwise analyses.

**Results:**

PSP and PD groups showed significant LC degeneration in the caudal subregion relative to controls. Mixed‐effect models revealed a significant interaction between disease‐group and apathy‐related correlations with LC degeneration (*β* = 0.46, SE [standard error] = 0.17, *F*(1, 35) = 7.46, *P* = 0.01), driven by a strong correlation in PSP (*β* = −0.58, SE = 0.21, *t*(35) = −2.76, *P* = 0.009). Across both disease groups, voxelwise analyses indicated that lower LC integrity was associated with worse cognition and higher apathy scores.

**Conclusions:**

The relationship between LC and nonmotor symptoms highlights a role for noradrenergic dysfunction across both PD and PSP, confirming the potential for noradrenergic therapeutic strategies to address transdiagnostic cognitive and behavioral features in neurodegenerative disease. © 2022 The Authors. *Movement Disorders* published by Wiley Periodicals LLC on behalf of International Parkinson and Movement Disorder Society

Parkinson's disease (PD) and progressive supranuclear palsy (PSP) result from distinct pathologies, yet they share many motor and nonmotor symptoms. These include executive dysfunction and neuropsychiatric features, with apathy.^1^, ^2^ Although the causes of cognitive and neuropsychiatric symptoms in these diseases are multifactorial, there is increasing evidence that noradrenergic dysfunction plays a prominent role in both.^3^, ^4^, ^5^

In PD and PSP, the noradrenergic locus coeruleus (LC) is a site of selective vulnerability, undergoing early and severe pathological changes.^6^, ^7^, ^8^ Diffuse projections enable the LC to release noradrenaline in widespread brain regions. The effects of noradrenaline at target regions include altering the gain of neurons and promoting reorganization of functional networks.^9^ Through these actions, noradrenaline influences many cognitive functions, facilitating attentional focus and flexible shifting between goal‐directed behaviors. In PD and PSP, we propose that vulnerability of LC neurons and consequent loss of noradrenergic projections have wide‐ranging impacts on cognition and behavior.^10^

Furthermore, given the possible contribution of noradrenergic deficits to cognitive and behavioral impairments in PD and PSP, they represent a promising treatment target that warrants further exploration. In tandem with progressing noradrenergic therapies, one needs to identify patients most likely to benefit from them. For example, individual differences in brain structure and neurochemistry may determine a patient's response to noradrenergic treatment, consistent with the nonlinear (U‐shaped) relationships that have been observed for dopaminergic and serotonergic interventions.^5^, ^11^, ^12^ In particular, we propose that an individual's need for—and response to—noradrenergic treatment depends on the state of his or her LC‐noradrenergic system.

Progress in noradrenergic therapeutics has been hindered by limited in vivo characterization of LC degeneration. The LC is small and elongated (~1 × 16 mm) with few neurons (~50,000 in humans), which challenges standard imaging techniques. Specialized magnetization‐transfer sequences that are sensitive to the intrinsic contrast generated by neuromelanin‐rich noradrenergic cells of the LC have been developed.^13^, ^14^ These correlate with the density of neuromelanin‐positive neurons in the LC,^15^ providing a biomarker of LC degeneration^16^ that confirms reductions in PD and PSP.^17^, ^18^ Such sequences are most sensitive at ultrahigh field 7 T magnetic resonance imaging (MRI),^19^, ^20^ providing both enhanced signal‐to‐noise ratio (SNR) and improved spatial resolution.

Here, we test the hypothesis that apathy and cognitive function correlate with the integrity of the LC in PD and PSP by exploiting ultrahigh field neuromelanin‐sensitive 7 T MRI. We took a transdiagnostic approach, based on the hypothesis that LC degeneration is a mechanism that explains individual differences in cognitive/psychiatric features, regardless of molecular pathology. The high resolution (0.08 mm^3^) of ultrahigh field imaging enabled the localization of degeneration to subregions of the LC. Given the prominent role the LC ‐noradrenergic system plays in cognition and goal‐directed behaviors, we predicted that clinical assessments of cognition and apathy would relate to LC integrity, with a rostrocaudal gradient of degeneration.

## Patients and Methods

### Participants

Sixty‐three participants aged 50 to 80 years were recruited, comprising 25 with idiopathic PD, 14 with PSP‐Richardson's syndrome, and 24 age‐ and sex‐matched healthy controls (HCs). Patients with idiopathic PD were recruited via the University of Cambridge Parkinson's Disease Research Clinic and the Parkinson's UK volunteer network and met the United Kingdom Parkinson's Disease Society Brain Bank criteria for PD diagnosis. Patients with PSP were recruited from the Cambridge University Centre for Parkinson‐Plus, with probable PSP‐Richardson's syndrome (Movement Disorder Society 2017 criteria). HCs were recruited from local volunteer panels. No controls were using psychoactive medications, and exclusion criteria for all participants included stroke, severe comorbidity, and contraindications to 7 T MRI. None of the patients had impulse control disorders, based on clinical impression or the Questionnaire for Impulsive‐Compulsive Disorders in Parkinson's Disease screen. Patients did not have dementia, based on Mini‐Mental State Examination (MMSE score >26)^21^ and clinical impression. All PD patients and 10 of 14 PSP patients were taking dopaminergic medications; levodopa equivalent daily dose (LEDD) was calculated. The study was approved by the local Cambridge Research Ethics Committees, and participants provided written informed consent.

### Clinical Assessments

Participants completed assessments of global cognition (MMSE, Addenbrooke's Cognitive Examination‐Revised [ACE‐R], and Montreal Cognitive Assessment [MoCA]) and self‐rated mood/behavior questionnaires: Apathy Scale (AS),^22^ Barratt Impulsiveness Scale (BIS),^23^ Hamilton Anxiety and Depression Scale (HADS, noting that several questions are probing apathy, not low mood), and REM Sleep Behavior Disorder Screening Questionnaire (RBDSQ). Higher scores on these questionnaires indicate more severe impairment. For patients, motor severity was assessed using the Movement Disorder Society Unified Parkinson's Disease Rating Scale (MDS‐UPDRS‐III). PSP patients additionally underwent the PSP Rating Scale (PSPRS). Higher scores reflect more severe motor symptoms. Patients underwent MRI and clinical assessments on their regular medications (see Table 1).

**TABLE 1:** Mean (standard deviation) for demographics and clinical assessments

	Descriptive			*P‐*values for pairwise tests		
	HC	PD	PSP	HC vs. PD	HC vs. PSP	PD vs. PSP
Age (y)	65.5 (5.5)	67.4 (7.4)	69.7 (7.7)	0.579	0.163	0.576
Education (y)	14.8 (3.1)	14.1 (2.3)	12.3 (2.8)	0.603	0.02	0.13
Male/female	13/11	18/7	8/6	0.196	0.859	0.345
MMSE	29.75 (0.53)	29.52 (0.65)	28.5 (1.74)	0.685	**<0.001**	0.007
MoCA	28.58 (1.44)	27.96 (1.88)	24 (3.94)	0.628	**<0.001**	**<0.001**
ACE‐R total	97.71 (3.25)	95.40 (3.61)	87.21 (7.17)	0.182	**<0.001**	**<0.001**
Apathy Scale	10.38 (5.25)	12.44 (5.43)	20 (9.49)	0.508	**<0.001**	0.003
BIS	55.71 (9.56)	58.18 (10.31)	63.86 (12.44)	0.692	0.064	0.248
HADS depression	2.83 (2.84)	4.24 (2.73)	7.43 (4.27)	0.273	**<0.001**	0.01
HADS anxiety	4.29 (3.53)	5.04 (3.16)	6.57 (3.2)	0.711	0.11	0.356
RBDSQ	–	5.48 (3.65)	3.07 (1.82)	–	–	0.027
Disease duration (y)	–	5 (3.05)	4.24 (2.68)	–	–	0.438
LEDD	–	644.3 (499.36)	323.57 (389.4)			0.038
UPDRS‐III	–	28.36 (11.98)	33.07 (6.96)	–	–	0.187
PSPRS	–	–	30.79 (9.11)	–	–	–

Group difference in sex was examined using χ^2^ test. A one‐way analysis of variance was used for group difference with post hoc Tukey*’s* honestly significant difference *P‐*values provided for pairwise comparisons. Significant *P‐*values (*P* < 0.0016, equivalent to *P* < 0.05, with Bonferroni correction for multiple comparisons of all tests on demographics and clinical assessments) are highlighted in bold font.

RBDSQ, disease duration, and UPDRS‐III were compared with independent sample *t* test between PD and PSP.

Abbreviations: HC, healthy control; PD, Parkinson's disease; PSP, progressive supranuclear palsy; MMSE, Mini‐Mental State Examination; MoCA, Montreal Cognitive Assessment; ACE‐R, Addenbrooke's Cognitive Examination‐Revised; BIS, Barratt Impulsiveness Scale; HADS, Hamilton Anxiety and Depression Scale; RBDSQ, REM Sleep Behavior Disorder Screening Questionnaire; LEDD, levodopa equivalent daily dose; UPDRS, Unified Parkinson's Disease Rating Scale; PSPRS, Progressive Supranuclear Palsy Rating Scale.

### MRI Acquisition

Participants underwent MR scan on a 7 T Magnetom Terra (Siemens, Erlangen, Germany) with a 32‐channel head coil (Nova Medical, Wilmington, MA, USA). The LC was imaged using a sensitive 3D magnetization transfer (MT) weighted sequence^19^ at high resolution (0.4 × 0.4 × 0.5 mm^3^), following the same protocol in previous studies.^5^, ^20^ A high‐resolution T1‐weighted structural image (0.7 mm isotropic) was acquired using MP2RAGE sequence with the UK7T Network harmonized protocol.^24^ Details of MRI parameters are provided in Supplementary Methods.

### LC Integrity Estimation

**FIG 1:**
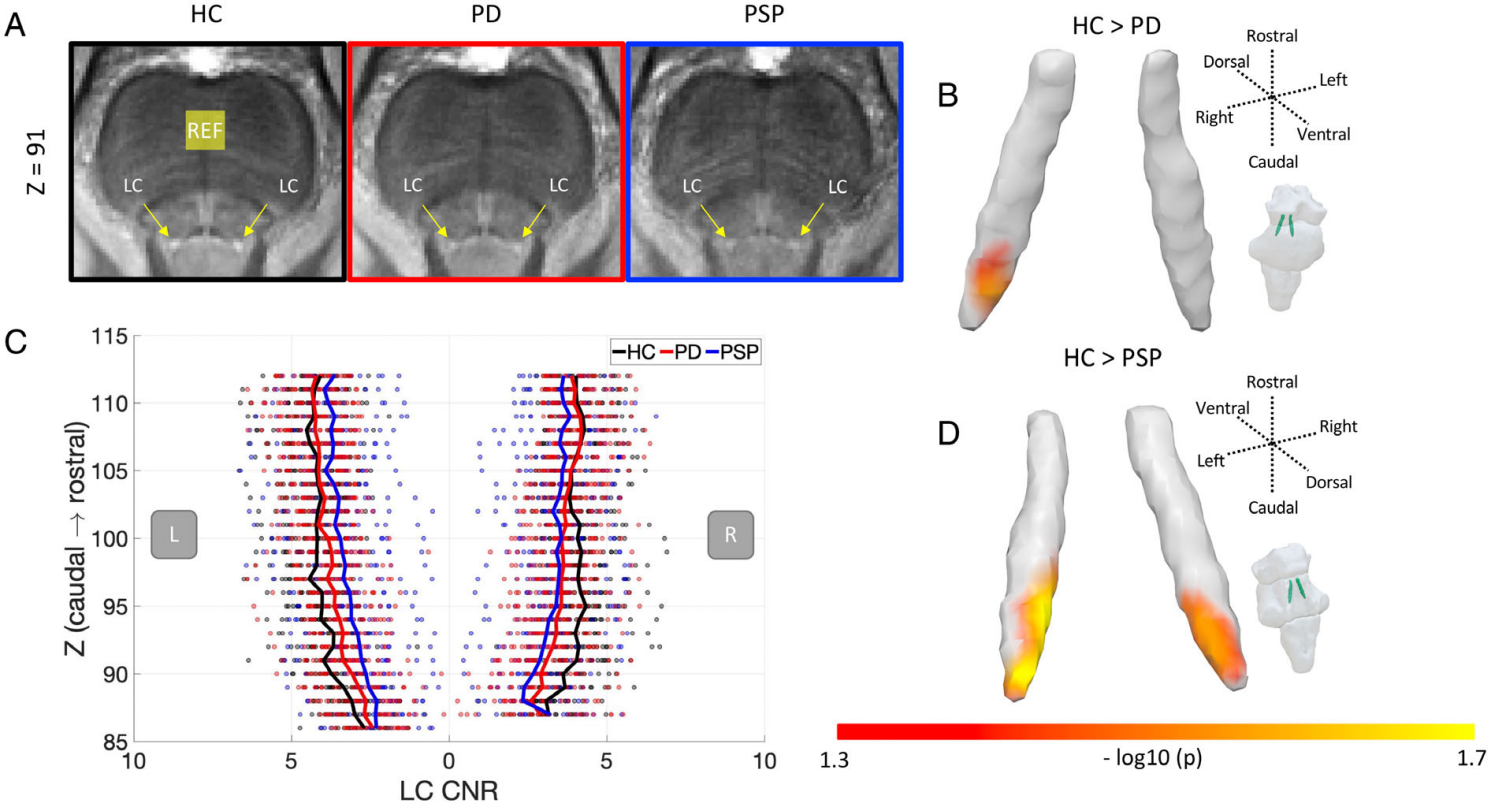
Comparisons of LC (locus coeruleus) integrity across the three groups. (**A**) Group‐averaged axial MT (magnetization‐transfer) images coregistered with the ICBM152 standard template where diminished LC (arrows pointing to) contrast can be observed in both PD and PSP (progressive supranuclear palsy) patients. (**B**) The rostrocaudal distributions (top: rostral, bottom: caudal) of LC CNR (contrast‐to‐noise ratio) calculated against a reference region (“REF” in A) in the pontine tegmentum area depicted a rostrocaudal gradient of the contrast reduction in disease groups. Voxelwise analyses further confirmed LC clusters with significant contrast reduction in (**C**) right caudal LC for PD and (**D**) bilateral caudal LC for PSP compared to controls (threshold free cluster enhancement, 10,000 permutations, family‐wise error [FWE]‐corrected *P* < 0.05). [Color figure can be viewed at wileyonlinelibrary.com]

To limit potential confounds in standard intensity‐based segmentation and increase the sensitivity, an atlas‐based segmentation approach was adopted to extract LC signal using a 5% probabilistic LC atlas in the standard space, generated from an independent sample of 29 older healthy volunteers. The MT images were coregistered to the high‐resolution (0.5 mm isotropic) ICBM152 2009b template following a T1‐driven, intra‐modality coregistration pipeline.^20^ The contrast‐to‐noise ratio (CNR) of the LC was computed against a 4 × 4 × 4.5 mm^3^ reference region placed at the central pons (Fig. 1A) using the difference between a given voxel and the mean intensity of the reference region divided by the standard deviation (SD) of the reference signals. This pontine reference region was chosen as a measurement source of noise due to its proximity to the LC. Although significant pontine tau pathology has been reported in PSP‐Richardson's syndrome,^25^ the MT signal did not differ between PSP patients and controls in the current study (*t*(36) = 0.47, *P* = 0.64), confirming this area as a suitable reference region. The independent LC atlas was applied to extract averaged CNR for each slice (for data visualization only, Fig. 1B), three equidistantly subdivided regions (rostral, central, and caudal), left and right LC, and the whole structure. The same LC mask was also used in the voxelwise analyses.

To ensure our results were not driven by varied registration accuracy or signal quality, we generated two indices for testing these factors. FreeSurfer (v6.0.0), *‐reconall* function with *‐highres* and *‐brainstem* options, was used for segmenting and estimating volumes of brainstem substructures from the MP2RAGE images to obtain individual pons masks. We then calculated pairwise dice similarity coefficients (DSCs) between individual coregistered (I) and standard (S) pons masks ($\mathrm{DSC}=2\left(I\cap S\right)/I+S$) to index registration accuracy. Signal quality was measured with SNR by calculating the division between the mean and the SD of the raw signal on coregistered MT images for subregions and the whole LC. We compared the group difference in SNR for rostral, central, and caudal LC and examined the subregion × group interaction using repeated measures one‐way analysis of variance (ANOVA). Group differences in DSC and pons volume were tested using one‐way ANOVA. Bonferroni correction was used for post hoc tests.

### Statistical Analyses

Statistical analyses were performed in R (version 3.6.1). We hypothesized that the LC degeneration occurred along the rostrocaudal axis. Group differences in LC CNR were therefore examined within three subregions (rostral, central, and caudal) using analysis of covariances (ANCOVAs), with regional contrast as the dependent variable, group as the fixed factor, and SNR and DSC as covariates of no interest. Post hoc Tukey's honestly significant difference tests were used for pairwise group comparisons. Group differences in demographics and clinical assessments were tested using χ^2^ test and one‐way ANOVA. Multiple comparisons across models were further corrected using Bonferroni‐adjusted *P*‐values in ANCOVAs for LC contrast (adjusted *P* = 0.017) and in ANOVAs for demographics and clinical questionnaires (adjusted *P* = 0.0016).

The relationships between LC contrast and individual clinical assessments were tested using mean LC CNR in patient groups with linear mixed‐effect models, defined in R formula syntax as follows: LC CNR ~ score × group + LC subregion + (1|subjects). The outcome corresponds to CNR values extracted from the whole, left, and right LC to account for potential lateralized effects. We included the scores on a particular clinical assessment, group (PD vs. PSP) and their interaction effect, as well as LC definition (whole vs. left vs. right) as fixed effects. Given the transdiagnostic hypothesis, our principal analyses combined data from all patients. The study was not planned to be strongly powered to separately test relationships between LC and clinical measures in each group, although exploratory group‐specific effects are presented. A random effect of subjects on the intercept was included to account for the repeated‐measures aspect of the LC CNR data. This model was then fitted separately for MoCA, ACE‐R, and AS. Bonferroni correction was adopted for the significance threshold across three main models (adjusted *P* = 0.017).

### Voxelwise Analyses

Voxelwise analyses provide a more specific and spatially accurate localization for the LC group differences and clinical correlations, as averaged CNR is insensitive to voxel‐by‐voxel variations in LC integrity. The coregistered CNR maps were smoothed with a 1‐mm full‐width half‐maximum Gaussian kernel and masked with the 5% 7 T LC atlas. Voxelwise analyses were performed using PALM (alpha119) in FSL (v6.0.0). A threshold free cluster enhancement (TFCE) method was adopted for cluster inference combined with permutation tests (10,000 iterations). Multiple comparisons across contrasts were corrected using *‐corrcon* function in PALM.^26^ The corrected family‐wise error (FWE) *P*‐value (<0.05) was used to determine significant clusters. The *–*log*p* function and a significance level of 1.3 was used for better visualization (−log_10_^(0.05)^ ≅ 1.3) as recommended.

Specific spatial patterns for group difference and relationships with clinical variables of voxelwise LC contrast were identified using general linear models. Group comparisons were performed using paired *t* tests, with two contrasts for determining the direction of the group difference. For clinical scores, voxelwise analysis was implemented only to test both positive and negative relationships for assessments that were related to the hypothesis (MoCA, ACE‐R, and AS). Analogous regression models included age and LEDD as covariates of no interest to test potential confounds, because age and dopaminergic status may both contribute to cognitive and behavioral impairments.

### Exploratory Analyses

To explore the potential correlation between asymmetries of LC contrast and motor symptoms, we created two variables for categorizing the side (left or right) of lateralization for LC atrophy (lower contrast) in each subregion and the more affected side (left or right) for motor symptoms by weighing scores for the left and right sides from the MDS‐UPDRS‐III (higher scores for more severe motor symptoms). χ^2^ Tests were used for examining the independency, and Cramer's V was used to calculate the correlation coefficient for two categorical variables. We also calculated two asymmetry indexes (AI) for motor symptoms and whole‐LC contrast, respectively. AI was defined as AI=right side values−left side values/right side values+left side values to represent the degree of asymmetry. Potential sex difference in LC CNR and the age effect on LC contrast were tested using *t* test and linear regression models, respectively. The relationships of LC contrast with demographics and other clinical assessments were also tested (see Supplementary Materials).

## Results

### Demographics and Clinical Assessment

Demographics and clinical data are summarized in Table 1. Groups were age‐ and sex matched. People with PSP had fewer years of education compared to controls, and they were more impaired on the MMSE, MoCA, ACE‐R, AS, and HADS‐depression assessments relative to both controls and PD. People with PD had higher LEDD and RBDSQ scores than PSP.

### Data Quality Assurance

The SNR differed between subregions (*F*(2, 120) = 13.02, *P* < 0.001). The effect was evident in the rostral LC (LC_rostral_ > LC_central_: *p*_bonf_ < 0.001; LC_rostral_ > LC_caudal_: *p*_bonf_ = 0.003), whereas SNRs of central and caudal LC did not differ (*p*_bonf_ = 0.53). There was a significant group effect (*F*(2, 60) = 4.68, *P* = 0.01), and post hoc tests showed that PD group had higher SNR than PSP patients (*p*_bonf_ = 0.02). There was no group by subregion SNR interaction (*F*(4, 120) = 2.22, *P* = 0.07). Furthermore, there was no group difference observed for pons volumes (*F*(2, 59) = 2.27, *P* = 0.11) or LC registration accuracy estimated using DSC (mean ± SD: 0.875 ± 0.02, range: 0.74–0.89, *F*(2, 60) = 1.39, *P* = 0.26), indicating that the contrast difference in the LC was not attributed to these potential confounds.

### LC Group Differences

LC integrity was assessed using an atlas‐based approach, as opposed to segmentation‐based methods where the structure is segmented manually or with a specific threshold. Including both LC SNR and DSC as covariates of no interest to control variability in data quality, a group effect was detected in the caudal subregion (*F*(2, 60) = 5.38, *P* = 0.007) that was driven by a difference between PSP and controls (*p*_bonf_ = 0.006) but not for PD versus controls (*p*_bonf_ = 0.24) or PD versus PSP (*p*_bonf_ = 0.37). There was no group effect for the whole‐LC (*F*(2, 60) = 2.62, *P* = 0.08) or other subregions (rostral: *F*(2, 60) = 1.22, *P* = 0.3; central: *F*(2, 60) = 2.73, *P* = 0.07).

Voxelwise analyses revealed clusters with significantly reduced contrast (TFCE, *P* < 0.05, FWE corrected) in both disease groups compared to controls (Table 2). Focal LC signal change was identified for PD patients in the right caudal LC (Fig. 1C, 46 voxels, 5.8 mm^3^), whereas PSP patients had extensive bilateral caudal damage in the LC (Fig. 1D, 207 voxels, 25.9 mm^3^). There was no group difference between PD and PSP by voxelwise analysis.

**TABLE 2:** TFCE clusters within the locus coeruleus for regression models and group comparisons (FWE‐corrected *P* < 0.05)

	Coordinates of local maxima (mm)			Voxel	Volume (mm^3^)	*t*
	*x*	*y*	*z*			
Regression						
No covariates						
MoCA	−3	−38.5	−22.5	95	11.875	4.02
	4.5	−39.5	−26.5	66	8.25	4.34
AS	−4.5	−39	−22.5	165	20.625	4.02
Age and LEDD covariates						
MoCA	4.5	−39.5	−26.5	82	10.25	4.13
	−4.5	−40	−26.5	78	9.75	4.01
AS	−4.5	−39	−22.5	42	5.25	3.27
	−5	−39.5	−26	23	2.875	2.92
	−2.5	−37.5	−18	11	1.375	3.32
Group comparisons						
HC > PSP	−5	−40	−27.5	124	15.5	3.88
					
	3	−38.5	−25	83	10.375	3.86
HC > PD	4.5	−38.5	−27	46	5.75	3.43

Models were tested using FSL's PALM and corrected across contrasts with 10,000 permutations. Standard coordinates in MNI space (mm^3^) are reported.

Abbreviations: TFCE, threshold free cluster enhancement; FWE, family‐wise error; MoCA, Montreal Cognitive Assessment; AS, Apathy Scale; LEDD, levodopa equivalent daily dose; HC, healthy control; PSP, progressive supranuclear palsy; PD, Parkinson's disease.

### LC Integrity and Clinical Assessments in Patients

The mixed‐effect models revealed a positive trend between MoCA and LC CNR (*β* = 0.43, SE = 0.21, *F*(1, 35) = 4.33, *P* = 0.04), which did not survive correction for multiple comparisons. There was no group interaction (*F*(1, 35) = 0.4, *P* = 0.53), suggesting the effect was comparable in both disease groups. The main effect of LC CNR on Apathy Score was not significant (*F*(1, 35) = 0.44, *P* = 0.51). We observed a significant interaction between group and Apathy Score on LC CNR (*β* = 0.46, SE = 0.17, *F*(1, 35) = 7.46, *P* = 0.01). Post hoc testing indicated that there was a significant negative relationship between apathy and LC CNR in the PSP group (*β* = −0.58, SE = 0.21, *t*(35) = −2.76, *P* = 0.009) but not in the PD group (*β* = 0.35, SE = 0.27, *t*(35) = 1.31, *P* = 0.2). This suggests that more apathetic PSP patients tended to have lower LC CNR. The ACE‐R was not related to LC CNR (*F*(1, 35) = 2.08, *P* = 0.16), and there was no significant interaction with group (*F*(1, 35) = 0.14, *P* = 0.71).

**FIG 2:**
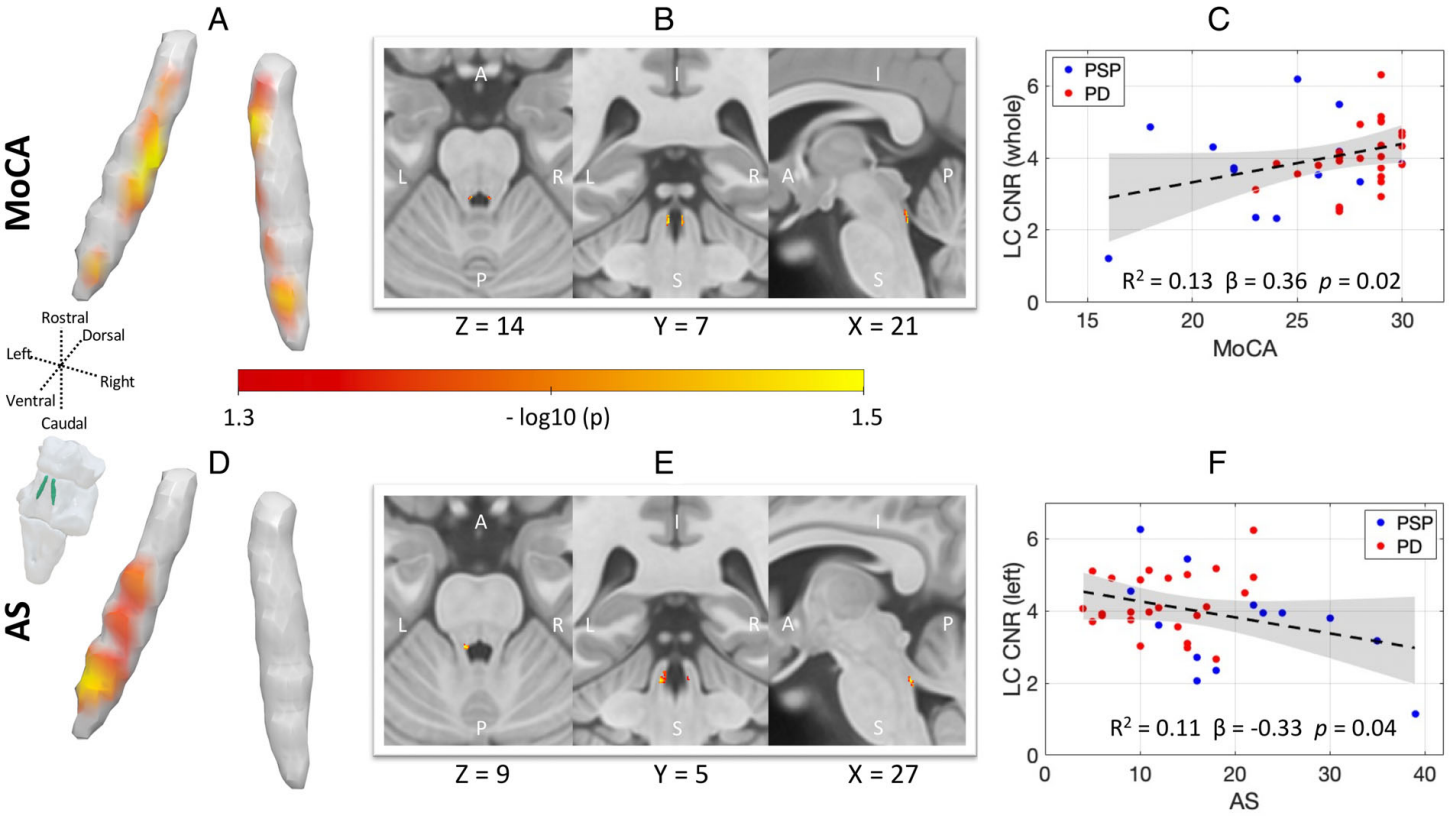
The role of LC (locus coeruleus) in global cognition and apathy. The MoCA (Montreal Cognitive Assessment) and Apathy Score (AS [Apathy Scale]) in PD and PSP (progressive supranuclear palsy) correlate with LC contrast. The spatial distributions within the LC contributing to global cognition and apathy are revealed from voxelwise regression models in 3D rendering and multiplanar views (MoCA: **A** and **B**, AS: **D** and **E**, threshold free cluster enhancement, 10,000 permutations, FWE [family‐wise error]‐corrected *P* < 0.05; age and LEDD [levodopa equivalent daily dose] were included as covariates of no interest). The linear relationships between atlas‐based LC CNR (contrast‐to‐noise ratio) and clinical scores over both disease groups were tested using mixed‐effect models, with individual variation illustrated by scatter plots for (**C**) MoCA and (**F**) AS, respectively (for visualization, fitted lines and confidence intervals were provided using linear regression models; see “Results” section for statistical tests using mixed‐effect linear models). [Color figure can be viewed at wileyonlinelibrary.com]

A voxelwise analysis would have advantages for detecting and localizing the effect of LC in cognition and apathy if there are localized effects or if the arbitrary division of the LC did not correspond to neuropathological divisions. As shown in Figure 2, the voxelwise results confirmed that bilateral LC clusters were positively correlated with MoCA scores: preserved LC integrity was associated with better cognitive performance. Consistently, the left‐central and caudal LC clusters were negatively correlated with Apathy Score: reduced LC integrity was associated with worse apathy (Table 2). Inclusion of age and LEDD as covariates of no interest did not meaningfully change the results of voxelwise analyses.

### Exploratory Analyses

The whole‐LC contrast did not differ by sex (*t*(62) = 0.7, *P* = 0.48). No linear or quadratic relationship was detected between LC contrast and age (linear: *F*(1, 61) = 0.84, *R* ^2^ = 0.01, *P* = 0.36; quadratic: *F*(2, 60) = 1.55, *R* ^2^ = 0.05, *P* = 0.16). There was no significant effect of lateralization on whole‐LC contrast (*F*(1, 60) = 3.802, *P* = 0.06), or group × lateralization interaction (*F*(2, 60) = 2.637, *P* = 0.08), although with regard to the weak trend we tested and confirmed a marginal left‐lateralized effect of LC contrast in PD (left > right: *p*_holm_ = 0.05). In PD patients, lower contrasts on one side of the rostral and central LC were correlated with more severe motor symptoms ipsilaterally (rostral: *χ* ^2^ = 5.24, *P* = 0.02, Cramer's *V* = 0.46; central: *χ* ^2^ = 3.74, *P* = 0.05, Cramer's *V* = 0.39; and caudal: *χ* ^2^ = 0.43, *P* = 0.51, Cramer's *V* = 0.13). There was a positive correlation between motor AI and LC AI in PD patients (*R* ^2^ = 0.22, *P* = 0.02), suggesting that the degree of lateralization was consistent between LC contrast and motor symptoms. Results from the mixed‐effect models indicate that the LC contrast was not associated with other clinical assessments (Supplementary Materials).

## Discussion

Using ultrahigh field 7 T magnetization‐transfer imaging, we confirm in vivo loss of LC integrity in PD and PSP, both disorders with previously well‐established LC pathology at *postmortem*. We confirm the regionally specific pattern of predominantly caudal LC changes in PD and PSP, which was associated with cognitive impairment and apathy. These findings highlight the importance of the LC ‐noradrenaline system in cognitive and behavioral features of these diseases. They demonstrate the utility of neuromelanin‐sensitive imaging at ultrahigh field to measure the LC in neurodegenerative disease, which we suggest as a biomarker for use in future clinical trials, where patients could be stratified according to LC integrity.^5^, ^20^

The main group‐wise effect of PD and PSP on the LC was most apparent in the caudal subregion. This rostrocaudal gradient has been noted in some neuropathological studies of PD,^27^ although not all,^28^ and is in keeping with more recent in vivo LC imaging showing a prominence of caudal changes in PD.^29^, ^30^ In contrast, Alzheimer's disease, Down's syndrome,^28^ and healthy aging^31^ have been associated with greater cell loss from the rostral LC. The caudal subregion may be especially vulnerable as it lies adjacent to the fourth ventricle, exposed to environmental insults from the cerebrospinal fluid.^32^ Also, the caudal LC receives projections from the vagal nerve via the nucleus of the solitary tract, which, speculatively, may make it more vulnerable to transmission of misfolded proteins from the periphery.^33^ Future studies would be required to establish the relationship to peripheral nervous system pathology.

In our study, the LC contrast did not differ between PD and PSP, although the spatial pattern of contrast reduction appeared more widespread in PSP compared to PD. Postmortem studies have confirmed significant neuronal loss of the LC in both diseases,^8^, ^34^ but evidence for more severe LC damage in PSP is inconsistent based on in vivo studies. Studies using a T1‐weighted sequence with manual LC segmentation have suggested a relative preservation in PSP patients compared to PD^17^, ^35^; however, the LC signal in PSP could be artificially inflated by manual segmentation as a result of potential volume loss. In contrast, multimodal imaging quantification of an LC voxel with peak intensity and its 10 connected neighbors, via a semiautomated approach, revealed more pronounced impairment in PSPRS than in PD, even though the structural difference in the LC did not significantly distinguish PSP from PD.^36^ This was consistent with our findings, where the potential volume effect was controlled for by applying an atlas‐based quantification pipeline. We suggest that the manual segmentation method needs to be carefully considered in future studies because the accuracy of LC integrity measurement can be confounded by volume differences.

In our PD and PSP participants, the relationship of LC signal to behavioral (apathy) and cognitive (MoCA) change was not confined to caudal regions but extended to mid‐ and rostral LC (Fig. 2). LC degeneration and reduced noradrenaline in neurodegenerative disease is linked with a higher risk of progression to dementia^4^, ^37^ and faster cognitive decline in older adults.^38^ The correlation of apathy to LC integrity—most clearly in the PSP group—is in keeping with extensive human and preclinical evidence linking goal‐directed behavior with the noradrenergic system.^39^, ^40^ Our results highlight that while cognitive and motivation deficits in neurodegenerative disease have multifactorial causes, the LC‐noradrenaline system plays a key role via its widespread modulation of target structures supporting these processes. Our study was not designed to test the contribution of LC to cognition and apathy in PD and PSP groups individually and may be underpowered to do so. Future studies may seek to determine whether the impact of LC degeneration differs according to the molecular pathologies of different parkinsonian disorders.

Cognitive decline and disorders of goal‐directed behavior, including the co‐occurrence of both apathy and impulsivity,^41^, ^42^ are common in both PD and PSP. The link we have shown between LC integrity and these nonmotor symptoms supports the rationale for noradrenergic therapies in selected patients. Preliminary work in PD suggests that the noradrenergic reuptake inhibitor atomoxetine can improve goal‐directed behavior in some people,^12^ where drug responsivity depends on LC integrity.^5^ The ability to detect localized changes in the LC, as demonstrated here in PD and PSP, suggests the possibility to stratify patients in future clinical trials of noradrenergic therapy of cognitive and behavioral symptoms of neurodegenerative disease.

The role of LC in motor control is less clear. We did not find an overall significant association between LC integrity and motor symptom severity in PD, although reduced signal in the rostral and central LC portions was associated with more severe motor symptoms ipsilaterally. This is consistent with recent evidence of lateralization of LC changes to the side of the body more affected by motor symptoms.^30^ Given LC ascending projections to ipsilateral motor regions,^43^ LC changes may affect motor pathways on the clinically defined most affected side. However, the association may be indirect, with ipsilateral propagation of α‐synuclein pathology leading to correlated pathology in both LC and motor systems, without a direct causal mechanism.^30^, ^44^

The current study exploited the benefit of ultrahigh field 7 T MRI for the assessment of the LC in vivo. The short imaging sequence^19^ was well tolerated by people with PD and PSP. For LC signal extraction, we advanced a cross‐modality coregistration pipeline and an atlas‐based approach. This maximizes spatial precision and avoids estimation biases that occur with manual or threshold‐based segmentation, which is critical in clinical cohorts where pathology‐related LC signal reduction is accompanied by increased segmentation failure and reduced spatial precision, leading to inflated contrast estimation. Potential confounds specific to LC imaging were also controlled and explicitly tested, including the consistency of noise level along the rostrocaudal axis and the effect of age. Moreover, the spatial localization of effects was further improved by voxelwise analyses that exploited the high resolution afforded by 7 T MRI. It is worth noting, however, that certain challenges remain when interpreting magnetization‐transfer imaging; for example, contrast is produced in regions that are low in neuromelanin, such as the periaqueductal gray.^45^ The advances we describe allow for more accurate LC quantification, and they also permit the localization of changes to LC subregions, which have previously been identified in postmortem studies.^28^ The histological validation of neuromelanin‐sensitive imaging^15^ provides additional evidence that the results we report here reflect the underlying pathological changes that have been observed at *postmortem*. Together, these findings pave the way for future clinical studies to investigate contributions of the LC‐noradrenaline system to the pathogenesis, progression, and treatment of neurodegenerative diseases.

## Conclusion

The LC is a site of selective vulnerability to neurodegenerative disease.^10^, ^16^ We provide in vivo confirmation of LC degeneration in PD and PSP and demonstrate its association with cognition and apathy. Ultrahigh field 7 T neuromelanin‐sensitive imaging provides a valuable tool for investigating nonmotor symptoms and has potential for stratifying patients in clinical trials of noradrenergic therapy.

## Author Roles

R.Y., C.O., T.W.R., L.P., and J.R. contributed to the conception and design of the study. M.M., C.O., C.R., F.H.H., P.S.J., N.H., M.M., C.R., R.A.B., and C.H.W.‐G. contributed to acquisition and analysis of data. R.Y., C.O., L.P., and J.R. contributed to drafting the text and preparing the figures.

## Supporting information


**APPENDIX S1**. Supporting Information

## Data Availability

The MT and MP2RAGE data used in this study are available for non‐commercial academic purposes upon request.
