# Supplementary material for: Locus Coeruleus Integrity from 7 T MRI Relates to Apathy and Cognition in Parkinsonian Disorders
Source: Mov Disord. 2022 May 16;37(8):1663–72. doi: 10.1002/mds.29072 (PMC9541468; doi:10.1002/mds.29072)
Supplement: Supplementary file 1 — APPENDIX S1. Supporting Information [file MDS-37-1663-s001.docx]

**Supplementary Materials**

**MRI parameters**

For the MT-weighted sequence, 112 oblique, high-resolution, axial slices were placed perpendicular to the long axis of the brainstem, covering both midbrain and pons. A train of 20 MT pulses at 6.72 ppm off resonance were applied followed by a turbo-flash readout (TE = 4.08 ms, TR = 1251 ms, flip-angle = 8°, voxel size = 0.4 x 0.4 x 0.5 mm^3^, 6/8 phase and slice partial Fourier, bandwidth = 140 Hz/px, no acceleration, 14.3%- oversampling, TA ~ 7 min). The MT sequence was repeated twice and averaged offline to enhance the signal-to-noise ratio. An additional scan without MT pre-saturation was acquired for registration purposes. A high resolution T1-weighted structural image was acquired using MP2RAGE sequence: TE = 2.58 ms, TR = 3500 ms, BW = 300 Hz/px, voxel size = 0.7 x 0.7 x 0.7 mm^3^, FoV = 224 x 224 x 157 mm^3^, acceleration factor (A>>P) = 3, flip angles = 5/2° and inversion times (TI) = 725/2150 ms for the first/second images.

**The relationship between LC CNR and other clinical assessments**

The mixed-effect model was tested for the relationship between LC CNR and other clinical assessments that were not related to our hypotheses (BIS, HADS, UPDRS-III and RBDSQ), defined in R formula syntax as follows: *LC CNR ~ score × group + LC subregion + (1 | subjects)*. For PSPRS, the model was only tested in PSP group.

Results from the mixed-effect models indicate that the LC contrast was not associated with UPDRS motor score (F(1,35) = 0.44, *p* = 0.51), RBDSQ (F(1,35) = 0.002, *p* = 0.96), BIS scores (F(1,35) = 3.25, *p* = 0.08), HADS anxiety (F(1,35) = 3.66, *p* = 0.06) or depression scores (F(1,35) = 2.01, *p* = 0.17). For people with PSP, the LC contrast was not correlated with the PSPRS (F(1,12) = 1.27, *p* = 0.28).
